# From isolation to inclusion: Understanding suicidality and school supports for LGB+ students in Japan

**DOI:** 10.1016/j.ssmph.2025.101836

**Published:** 2025-06-27

**Authors:** Adam O. Hill, Noriyo Kaneko, Natalie Amos, Adam Bourne, Masachika Yamashiro, Jami Jones, Stuart Gilmour

**Affiliations:** a School of Medicine, Nagoya City University, Nagoya, Japan; b Australian Research Centre in Sex, Health and Society, La Trobe University, Melbourne, Australia; c Kirby Institute, UNSW Sydney, Australia; d Graduate School of Public Health, St. Luke's International University, Tokyo, Japan

**Keywords:** Suicide attempt, Suicide prevention, LGB+ youth, Sexual and gender minorities, Japan, Educational settings, LGBT-inclusive bullying policies, School-based LGBT alliances

## Abstract

**Introduction:**

Youth suicide rates in Japan are among the highest globally, highlighting the urgent need for targeted prevention strategies. Within this context, schools can offer significant protective factors by shaping both identity development and mental health outcomes, but such protective effects have not examined in Japanese LGB+ populations. To address this research gap, this examined correlates of suicidal ideation and suicide attempts among LGB+ students in the past 12 months in Japanese educational settings.

**Methods:**

Multivariable logistic regression examined associations between recent suicidal ideation and suicide attempt and experiences of sexuality or gender-based harassment, exclusion, and school support structures among 2,095 LGB+ students aged 15-25 years in Japan from an anonymous online survey.

**Results:**

Results revealed high rates of suicidal ideation (32.3 %) and attempts (8.8 %) in the past 12 months. Sexuality and gender identity-based harassment, exclusion and hearing negative remarks about sexuality in educational settings were linked to higher suicidal ideation and suicide attempts. Supportive LGBT clubs and alliances and LGBT-inclusive anti-bullying policies at educational settings were associated with lower rates of suicidal ideation

**Conclusion:**

The findings highlight the urgent need for school-based interventions to mitigate the elevated risk of suicidality among LGB+ youth in Japan. Establishing safe and supportive educational environments may help reduce the heightened suicide risk faced by this vulnerable population

## Highlights

• High suicidal ideation (32.3%) and attempts (8.8%) in the past year among LGB+ students.
• Harassment, exclusion, and negative language based on sexuality associated with suicidality
• LGBT-inclusive bullying policies and supportive clubs associated with lower suicidal ideation
• Despite this, only 11 % of schools have such policies, and 15 % have a club/alliance
• Findings highlight urgent need for LGB + suicide prevention in Japanese educational settings

## 1 Introduction

Suicide is the fourth leading cause of death among young people worldwide, with increasing rates among youth in many countries (World Health Organization, 2021). Japan, in particular, has one of the highest youth suicide rates globally, with suicide being the leading cause of death among people aged 15–24 years. Recent data indicate that Japan's youth suicide rate has reached record levels (Ministry of Health et al., 2024), placing among the highest worldwide (World Health Organization. WHO, 2018; The Nippon Foundation) and indicating a growing mental health crisis among Japanese youth, exacerbated by societal pressures and limited mental health resources (The Nippon Foundation; Inoue et al., 2024). Despite these figures regarding youth suicide in Japan, there is a notable lack of national research focusing specifically on lesbian, gay, bisexual, pansexual, asexual and questioning (LGB+) youth, leaving critical gaps in understanding their unique experiences and needs (Hatzenbuehler, 2011).

Globally, LGB+ youth experience disproportionately high rates of suicidal ideation and suicide attempts compared to their heterosexual peers (Haas et al., 2011; King et al., 2008; Marshal et al., 2011; Skerrett et al., 2015). These disparities can be explained in part by minority stress theory, which posits that stigma, discrimination, and lack of support elevate stress levels and adversely affect mental health (Meyer, 2003). In Japan, despite limited data, LGBT youth under 20 years old report very high levels of suicidal ideation and suicide attempts, with 48 % experiencing suicidal thoughts and 14 % having attempted suicide in the past 12 months (ReBit, 2022). Educational settings often amplify these stressors, where LGB+ youth frequently encounter harassment, exclusion, and insufficient institutional support (Amos et al., 2024; Bucchianeri et al., 2016; Eisenberg et al., 2015; Martin-Storey & August 2016).

It is of note that although this study uses a cross-sectional design, we use the terms ‘risk’ and ‘protective’ factors in line with established usage in suicidality and minority stress research in Japan and internationally, where such terms describe associations with recent outcomes rather than causal predictors (Hatzenbuehler & Keyes, 2013; Hill et al., 2020, Hill et al., 2022). A variety of factors contribute to the elevated suicide risks faced by LGB+ youth. Internationally, harassment and bullying based on sexual orientation or gender identity are associated with higher odds of suicidal ideation and attempts (Birkett et al., 2009; Bouris et al., 2016; Hill et al., 2022). Feelings of exclusion and exposure to negative language about LGBT individuals may exacerbate isolation and hopelessness, further heightening suicide risks (Kosciw et al., 2009, 2020; Russell & Fish, 2016). Gender identity is also a significant contributor to the increased risk of suicidal ideation and suicide attempts among LGBT youth (Grossman & D'Augelli, 2007). To accurately capture the specific experiences of LGB+ cisgender youth in Japan, particularly within educational settings, this study focuses exclusively on this population. Trans and gender diverse youth face unique risk factors, such as challenges accessing gender-appropriate facilities at school and experiences of dead-naming and misgendering, which require separate examination (Hill et al., 2021; Hill, Cook, et al., 2023). By focusing on LGB+ cisgender students, this study aims to provide a more precise understanding of the unique factors contributing to their increased risk of suicidal ideation and suicide attempts. Findings on trans and gender diverse participants from the Writing Themselves in Japan (WTIJ) survey will be presented separately.

In addition to understanding risk factors, it is essential to consider the protective factors associated with lower odds of suicidal ideation and suicide attempts among LGB+ youth. Protective factors in educational settings, such as LGBT-inclusive anti-bullying policies and supportive LGBT groups and clubs or alliances, are associated with lower odds of suicidality through fostering safer school climates and providing social support (Marx & Kettrey, 2016). For instance, research in the United States indicates that LGBT youth in areas with inclusive anti-bullying policies are significantly less likely to attempt suicide compared to those in areas without such policies (Hatzenbuehler & Keyes, 2013).

In Japan, the absence of comprehensive anti-discrimination laws inclusive of sexual orientation and gender identity leaves LGBTQA+ individuals vulnerable to discrimination in education, employment, and other settings (Taniguchi et al., 2024). While the ‘Act on the Promotion of Citizens’ Understanding of Diversity of Sexual Orientation and Gender Identity,’ enacted in June 2023, represents a step toward awareness (Human Rights Watch, 2023), it lacks enforceable measures to prohibit discrimination or penalize violations. This legislative gap highlights Japan's limited legal protections for LGBT+ people compared to other developed nations. Within schools, these policy deficiencies, coupled with insufficient teacher training, contribute to bullying and a lack of supportive environments for LGBT students (Knight, 2016). Despite governmental efforts to address youth suicide through the *General Principles of Suicide Prevention Policy* (GPSPP) (Matsumoto et al., 2024), rates among those under 20 have remained persistently high (Matsumoto et al., 2024), reflecting the need for targeted measures that specifically address the vulnerabilities of LGBTQA+ youth.

This study aims to fill critical gaps in the literature by identifying risk and protective factors associated with suicidal ideation and suicide attempts over the past 12 months among LGB+ students in Japan. Specifically, this study examines the associations between experiences such as sexuality or gender identity-based harassment, exclusion, exposure to negative language regarding sexuality, and the presence of supportive school resources like LGBT-inclusive bullying policies and supportive clubs and suicidal ideation and suicide attempts among LGB+ youth in educational settings. Findings will contribute to a better understanding of LGB+ youth experiences and inform the development of effective, culturally sensitive interventions within educational settings and public health frameworks.

## 2 Methods

### 2.1 Sample and procedure

Data for this study were obtained from the Writing Themselves in Japan (WTIJ) survey, an anonymous, cross-sectional online survey examining the health and wellbeing of 5323 individuals aged 15–25 years who identified as lesbian, gay, bisexual, pansexual, transgender or gender diverse, queer, asexual (LGBTQA+) and resided in Japan. Data collection occurred between 31st May and July 28, 2024. Eligibility criteria required participants to be between the ages of 15 and 25, identify as LGBTQA+, and reside within Japan. Participants confirmed their eligibility by reading a detailed study description and providing consent via two mandatory checkboxes. Parental consent was waived for those under 18.

The majority of participants were recruited through paid social media advertisements that featured promotional animations and images. Supplementary recruitment was conducted through partnerships with Japanese LGBTQA+ organizations and networks, which promoted the survey online and distributed promotional cards. Participants were not compensated financially.

To ensure data accuracy, respondents under the age of 15 and those over 25 (n = 388), and junior high school students aged 15 years (n = 35) were excluded, resulting in a final sample after data cleaning of 5323 participants who completed the survey. For this analysis, we focused specifically on cisgender respondents enrolled in educational institutions (n = 2095), whose gender identity matched their sex assigned at birth, and who provided responses on recent suicidal ideation and attempts.

Ethical approval for the WTIJ study was obtained from the Human Research Ethics Committee of St Luke's International University.

## 3 Materials

The WTIJ survey was adapted from the “Writing Themselves In 4” survey conducted in Australia, designed with input from a Community Advisory Board and Youth Advisory Boards. For the Japanese context, the WTIJ survey was modified in collaboration with LGBTQA+ experts, including researchers, community advisors, and youth advisors with lived experience. A bilingual research team translated the survey into Japanese and backtranslated it into English for accuracy. The survey was available in Japanese and English.

**Gender**: Participants selected the gender that best described them from a list of 18 options, with the option to skip. Only cisgender male and female participants were included in this study.

**Sexuality:** Sexual orientation options included “gay,” “lesbian,” “bisexual,” “pansexual,” “queer,” “asexual,” “heterosexual/straight,” “prefer not to answer,” “prefer not to have a label,” “unsure/questioning,” and “something different.” Due to small sample sizes, responses in the “queer,” “prefer not to have a label,” and “something different” categories were grouped together. Cisgender heterosexual/straight individuals were included, reflecting Japan's unique cultural context, where same-gender attraction and sexual behaviours may not correspond to Western sexual identity labels. Specifically, Japan has a tradition of same-gender behaviours that do not necessarily conform to Western notions of sexual identity, allowing cisgender individuals to identify as heterosexual while still engaging in or being attracted to same-gender partners (Kazama & Kawaguchi, 2010; McLelland, 2000). Moreover, all participants consented at the survey's outset, confirming their LGBTQA+ identity, with no incentives for participation in the survey.

**Demographics:** Age groups were categorized as 15–17, 18–20, and 21–25 years. Participants were asked to report their age, with options ranging from ‘13 years old’ to ‘26 years or above’. Those who selected an age outside the eligible range (13, 14, or 26+) were automatically screened out of the survey and prevented from re-entering using a browser cookie. Participants who selected age 15 were then asked an additional question to confirm their current educational level. Those who indicated they were enrolled in junior high school were excluded from the final dataset. This decision was based on the ethical guidelines of St Luke's International University ethics committee, which required that all participants must have completed compulsory education (junior high school in Japan) or be currently enrolled in secondary school (high school) (typically ages 15–18). In Japan, junior high school is considered separate from secondary school and is part of the compulsory education system. As such, junior high school students aged 15 were not eligible to participate. A total of 35 participants aged 15 who were enrolled in junior high school were excluded from the final sample. We acknowledge that this may limit the generalizability of findings to younger adolescents, who may also be at elevated risk for suicidality (Hill et al., 2021; Patel, 2022).

Residential location: Upon peer-review, we ran robustness checks to assess whether the main associations remained stable when using a different way of categorisation. The original model included a four-category urban–rural variable (capital city, inner and outer suburbs, regional city or town, rural/remote). We then re-estimated the models using a regional variable based on Japan's seven major geographic blocks. Key predictors, such as gender identity, harassment, exclusion, and school-based LGBT clubs or alliances, showed consistent directionality and significance across both models. For example, the AOR for harassment and suicidal ideation was 1.45 (95 % CI: 1.11–1.89, p = 0.007) in the original model and 1.42 (95 % CI: 1.10–1.85, p = 0.006) in the regional model. Model fit also improved slightly based on information criteria (AIC = 2581.2, BIC = 2790.3). These values were lower than those obtained from the original model using the urban-rural variable, indicating a better fit. Additionally, multicollinearity decreased, with the average VIF declining from 2.07 to 2.00. Importantly, the association between LGBT bullying policy and suicidal ideation became statistically significant in the revised model, changing from 0.68 (95 % CI: 0.46–1.00, p = 0.050) to 0.66 (95 % CI: 0.45–0.97, p = 0.036). This may reflect the removal of confounding or multicollinearity introduced by the urban-rural variable, which may have obscured the independent association in the original model. Additionally, the regional classification aligns more closely with how residential location is typically operationalised in Japanese public health research, offering both conceptual and practical advantages for interpretation and policy relevance.

Ethnicity was recorded as “Japanese” or “other/mixed,” and participants’ educational status and employment engagement were also noted.

**Mental Health Measures:** Participants were asked about recent suicidal ideation and attempts in the past 12 months. Suicidal ideation was assessed with, “Have you had thoughts about suicide, wanting to die, or thoughts about ending your life?” For suicide attempts, the question was, “Have you attempted suicide or tried to end your life?” Responses included “no,” “yes, in the past 12 months,” “yes, more than 12 months ago,” and “prefer not to answer.” Those who selected “prefer not to answer” were excluded from the analysis. Contact information for mental health support was provided before and after these questions, with a message encouraging participants to skip if uncomfortable. A total of 146 (6.1 %) cisgender participants in enrolled in an education institution skipped this section and were excluded from the analysis; additionally, 37 (1.5 %) selected ‘prefer not to answer’ for suicidal ideation and 59 (2.5 %) chose ‘prefer not to answer’ for suicide attempts and were excluded from the respective analyses.

**Disclosure and Religiosity:** Participants indicated if they had disclosed their sexual or gender identity to friends or family, with response options grouped for analysis. Family or household religiosity was assessed with a “yes” or “no” question.

**Experiences of Harassment and Exclusion:** The survey inquired about verbal, physical, or sexual harassment or assault based on sexuality or gender identity within the past year. Responses were categorized as “any verbal, physical or sexual harassment or assault” or “none.” Participants were also asked if they felt excluded or left out due to their sexuality or gender identity in the past year, with responses categorized as “yes” or “no” for analysis.

**Comfort at educational setting**: participants were asked, “During the past 12 months have you felt unsafe or uncomfortable at your education setting because of your sexuality, gender identity and/or gender expression?” Responses were “no”, “yes, due to my sexuality,” “yes due to my gender identity or gender expression.” Responses were grouped into “no” and “yes, due to sexuality or gender identity/expression” for analyses.

**LGBT-specific school bullying policies:** participants were first asked, “Does your educational setting have a policy or program for addressing bullying, harassment, and assault?” with the response options “no,” “yes,” and “don't know.” Those who responded “yes” were given a follow-up question: “Does this specifically mention (select all that apply):" with options “sexuality,” “gender,” “all aspects of LGBT,” “no aspects of LGBT,” and “don't know.” Responses were coded as follows: Participants who initially answered “yes” to the policy question and selected “sexuality,” “gender identity,” or “all aspects of LGBT” in the follow-up were categorized as “yes” for LGBT-specific policy. If participants answered “yes” to the policy question but selected “no aspects of LGBT” in the follow-up, or if they initially responded “no” to the policy question, they were recoded as “no” for LGBT-specific policy. Additionally, participants who responded, “don't know” to either the initial question or the follow-up were recoded as “don't know.”

**Supportive LGBT clubs/alliances:** participants were asked, ‘Does your education setting have a supportive club for LGBT students?’ with the response options “no,” “yes,” and “don't know."

**Exposure to negative remarks about sexuality**: participants were asked, “In the past 12 months, how often have you heard any negative remarks regarding sexuality (e.g., “that's so gay”), whether or not they were directed at you, in your educational setting?” Response options included “never,” “rarely,” “sometimes,” and “frequently."

## 4 Statistical analyses

In this study, statistical analyses were conducted using Stata (Version 16 SE, StataCorp, College Station, TX, USA). Descriptive statistics were used to summarize all variables. Multivariable logistic regression models were employed to examine the associations between predictor (independent) and outcome (dependent) variables. The independent variables of interest included experiences of verbal, physical, or sexual harassment or assault related to sexuality or gender identity within the past 12 months, instances of exclusion based on sexuality or gender identity in the past year, family or household religiosity, comfort with being LGBTQA+ at education setting, presence of bullying policy specific to LGBTQA+ students or club supportive of LGBTQA+ people at their education setting. Demographic factors, including age, gender, sexual orientation, ethnicity, educational setting, employment status, and residential area, were included in the models as control variables to account for their potential confounding effects. These variables were not the primary focus of the study but were adjusted for to isolate the relationships between predictors of interest and the outcomes. The dependent variables analysed were suicidal ideation and suicide attempts reported in the previous 12 months.

Univariable logistic regressions were initially conducted to analyse unadjusted associations between each predictor and the outcome variables. For each model, unadjusted odds ratios (ORs), 95 % confidence intervals (CIs), and p-values were reported. Following this, two multivariable logistic regression models were constructed to investigate the relationships between predictors and outcomes while controlling for other factors. Variables with p-values less than 0.250 in univariable models were included in the multivariable models to account for variables that may gain significance when adjusted for other factors, following standard practices to avoid excluding potentially relevant predictors prematurely (Mickey & Greenland, 1989). Variance inflation factors (VIFs) were examined to assess collinearity, with all VIFs below 1.5 (mean VIF = 1.2), indicating no issues with collinearity. Adjusted odds ratios, 95 % confidence intervals, and p-values were presented for the multivariable models.

## 5 Results

### 5.1 Sample characteristics

Table 1 shows descriptive statistics for participants with complete data on all independent variables used in the regression models. The sample consisted mainly of individuals aged 18–20 (59.6 %), followed by those aged 21–25 (24.4 %) and 15–17 (16.0 %). In terms of sexual orientation, gay participants comprised the largest group (29.3 %), with bisexual participants making up 24.6 %, followed by lesbian (14.1 %), pansexual (9.4 %), and smaller proportions identifying as heterosexual (6.1 %), asexual (3.3 %), unsure/questioning (6.0 %), or something else (7.3 %).

**Table 1:** Descriptive statistics for all study variables (N = 2, 283).

	Number (n)	%
**Age group (years)**		
15–17	355	15.5
18–20	1378	60.4
21–25	550	24.1
**Sexual orientation**		
Lesbian	319	14.0
Gay	681	29.8
Bisexual	574	25.1
Pansexual	203	8.9
Heterosexual	133	5.8
Asexual	74	3.2
Unsure	136	6.0
Something else	163	7.3
**Gender identity**		
Cisgender man	962	42.1
Cisgender woman	1321	57.9
**Ethnic background**		
Japanese	2128	93.2
Other/mixed	155	6.8
**Current educational setting**		
secondary school (high school)	448	19.6
university	1462	64.0
2-year university/vocational school	242	10.6
Other	131	5.7
**Currently engaged in employment**		
No	730	32.0
yes	1553	68.0
**Family or household Religious**		
No	1924	84.3
Yes	359	15.7
**Residential location**		
Chubu	302	13.2
Chugoku & Shikoku	166	7.3
Hokkaido	82	3.6
Kanto	1006	44.1
Kansai	433	19.0
Kyushu & Okinawa	167	7.3
Tohoku	127	5.6
**Disclosed sexuality or gender identity to family**		
No	1151	50.4
a few/some	463	20.3
most/all	154	6.7
Not applicable	515	22.6
**Disclosed sexuality or gender identity to friends**		
No	449	19.7
a few/some	1357	59.4
most/all	214	9.4
Not applicable	263	11.5
**Any verbal, physical or sexual harassment in past 12 months based on sexuality or gender identity**		
no	1910	83.7
yes	373	16.3
**Felt deliberately left out or excluded in past 12 months based on sexuality or gender identity**		
no	2140	93.7
yes	143	6.3
**Felt uncomfortable at education setting due to sexuality or gender identity in past 12 months**		
No	1583	69.3
Yes	700	30.7
**School has LGBT bullying policy**		
No	536	23.5
Yes	256	11.2
Don't know	1491	65.3
**Supportive LGBT alliance at educational institution**		
No	1136	49.8
Yes	342	15.0
Don't know	805	35.3
**Heard negative remarks regarding sexuality even if was not directed at you**		
Never	856	37.5
Rarely	652	28.6
Sometimes	659	28.9
Frequently	116	5.1
**Suicidal ideation in past 12 months**		
No	1428	67.8
Yes	677	32.2
**Suicide attempt in past 12 months**		
No	1827	91.5
Yes	170	8.5

Three-fifths (59.2 %) identified as cisgender women, and 40.8 % as cisgender men. Most participants reported Japanese ethnicity (93.1 %), the majority were attending university (63.7 %), with others in secondary school (19.8 %), 2-year university or vocational school (10.8 %), or other educational settings (5.8 %). Employment was reported by two-thirds (67.2 %) of participants.

Few participants (15.2 %) reported having a religious family or household background. Two-fifths (39.9 %) lived in regional cities or towns, one-quarter (25.6 %) in rural or remote areas, 23.6 % in outer suburban areas, and 10.9 % in inner suburban areas of capital cities.

Half (50.6 %) of participants had not disclosed their sexuality or gender identity to family, while 20.2 % had shared with some family members, and 6.7 % with most or all. Disclosure to friends was more common, with two-thirds (60.0 %) sharing with a few or some friends and 9.2 % with most or all friends.

### 5.2 Discomfort, negative language regarding sexuality and harm

In terms of harassment, 16.2 % experienced verbal, physical, or sexual harassment based on sexuality or gender identity in the past 12 months, and 6.2 % felt deliberately excluded. Regarding exposure to negative remarks about sexuality, even if not directed personally, 38.3 % reported never hearing such remarks, 28.6 % indicated they heard them rarely, 28.3 % indicated they heard them sometimes, and 4.8 % indicated they heard them frequently. Additionally, one-third (30.5 %) reported feeling uncomfortable in an educational setting due to their sexuality or gender identity.

### 5.3 LGBT bullying policy and supportive LGBT club or alliance

One-quarter (23.8 %) of participants reported that their school did not have a specific anti-LGBT bullying policy, while 11.1 % reported that such a policy was in place. Two-thirds (65.1 %) were unsure about whether their school had an LGBT-inclusive bullying policy.

One-half (49.9 %) indicated that their institution did not have an LGBT alliance, 15.0 % reported having one, and one-third (35.1 %) were uncertain about its existence.

### 5.4 Suicidal ideation and attempts

One-third (32.3 %) of participants reported experiencing suicidal ideation and 8.8 % a suicide attempt in the past 12 months.

## 6 Regression analyses

### 6.1 Suicidal ideation in the past 12 months

Table 2 presents adjusted odds ratios (AORs) for factors associated with suicidal ideation in the past 12 months. Compared to cisgender men, cisgender women had higher odds of suicidal ideation (AOR = 2.01, 95 % CI: 1.41–2.87, p = 0.000). Participants who identified as heterosexual had lower odds of suicidal ideation than those who identified as gay (AOR = 0.56, 95 % CI: 0.32–0.98, p = 0.042). Educational setting also showed an association. Compared to secondary school students, those attending 2-year universities or vocational schools reported lower odds of suicidal ideation (AOR = 0.62, 95 % CI: 0.38–0.99, p = 0.044). Regional variation was observed among residential location, with participants in the *Chugoku* and *Shikoku* region having significantly higher odds of suicidal ideation compared to those in *Kanto* (Tokyo and surrounding metropolitan areas) (AOR = 1.58, 95 % CI: 1.10–2.27, p = 0.014).

**Table 2:** Regression results for suicidal ideation in past 12 months (N = 2107).

	Suicidal ideation		Univariable regression		Multivariable regression	
Variable	Number (n)	(%)	OR (95 % CI)	P-value	AOR (95 % CI)	P-value
**Age group (years)**						
15–17	148	41.6	REF		REF	
18–20	421	31.9	0.66 (0.52–0.84)	0.001	0.98 (0.64–1.50)	0.938
21–25	149	27.2	0.53 (0.40–0.70)	0.000	0.78 (0.49–1.26)	0.315
**Sexual orientation**						
Gay	184	28.3	REF		REF	
Lesbian	116	37.5	1.53 (1.15–2.03)	0.004	0.72 (0.45–1.15)	0.166
Bisexual	164	30.1	1.09 (0.85–1.40)	0.488	0.71 (0.49–1.03)	0.074
Pansexual	78	37.1	1.50 (1.08–2.08)	0.015	0.84 (0.52–1.35)	0.470
Heterosexual	30	21.9	0.71 (0.46–1.10)	0.129	0.56 (0.32–0.98)	0.042
Asexual	34	45.9	2.16 (1.32–3.51)	0.002	1.28 (0.68–2.39)	0.446
Unsure	53	39.8	1.68 (1.14–2.48)	0.008	1.08 (0.64–1.83)	0.771
Something else	57	36.3	1.45 (1.00–2.09)	0.049	0.87 (0.53–1.44)	0.591
**Gender identity**						
Cisgender man	239	26.4	REF		REF	
Cisgender woman	478	36.4	1.60 (1.33–1.92)	0.000	2.01 (1.41–2.87)	0.000
**Ethnic background**						
Japanese	663	32.0	REF		REF	
Other/mixed	55	35.7	1.18 (0.84–1.66)	0.346		
**Current educational setting**						
Secondary school (high school)	182	41.6	REF		REF	
University	420	29.7	0.59 (0.48–0.74)	0.000	0.85 (0.56–1.27)	0.428
2-year university/vocational school	68	28.3	0.56 (0.40–0.78)	0.001	0.62 (0.38–0.99)	0.044
Other	46	36.2	0.80 (0.53–1.20)	0.281	1.05 (0.62–1.76)	0.861
**Currently engaged in employment**						
No	268	36.9	REF		REF	
yes	449	30.1	0.73 (0.61–0.89)	0.001	0.89 (0.71–1.11)	0.301
**Family or household Religious**						
No	606	32.2	REF		REF	
Yes	111	33.0	1.04 (0.81–1.33)	0.753		
**Residential location**						
Kanto	293	30.3	REF		REF	
Chubu	95	32.9	1.13 (0.85–1.49)	0.401	1.13 (0.83–1.52)	0.437
Chugoku & Shikoku	63	39.9	1.53 (1.08–2.16)	0.017	1.58 (1.10–2.27)	0.014
Hokkaido	26	36.1	1.30 (0.79–2.15)	0.301	1.20 (0.70–2.07)	0.498
Kansai	132	32.0	1.09 (0.85–1.39)	0.515	1.12 (0.86–1.46)	0.403
Kyushu & Okinawa	42	27.6	0.88 (0.60–1.29)	0.509	0.78 (0.53–1.16)	0.223
Tohoku	44	37.9	1.41 (0.94–2.10)	0.093	1.45 (0.94–2.23)	0.094
**Disclosed sexuality or gender identity to family**						
None	355	31.7	REF		REF	
a few/some	161	35.9	1.21 (0.96–1.52)	0.111	1.16 (0.89–1.50)	0.277
most/all	55	36.7	1.25 (0.88–1.78)	0.220	1.36 (0.89–2.09)	0.155
Not applicable	146	29.3	0.89 (0.71–1.12)	0.333	0.91 (0.66–1.25)	0.563
**Disclosed sexuality or gender identity to friends**						
None	139	32.0	REF		REF	
a few/some	439	33.0	1.04 (0.83–1.32)	0.720	0.97 (0.74–1.27)	0.829
most/all	69	33.7	1.08 (0.76–1.53)	0.681	1.05 (0.68–1.63)	0.814
Not applicable	68	27.8	0.82 (0.58–1.15)	0.246	0.95 (0.59–1.55)	0.842
**Any verbal, physical or sexual harassment in past 12 months based on sexuality or gender identity**						
No	569	30.8	REF		REF	
yes	147	40.7	1.54 (1.22–1.94)	0.000	1.41 (1.07–1.86)	0.013
**Felt deliberately left out or excluded in past 12 months based on sexuality or gender identity**						
No	647	31.2	REF		REF	
yes	66	47.8	2.02 (1.43–2.86)	0.000	1.57 (1.05–2.36)	0.029
**Feel uncomfortable at educational setting due to sexuality or gender identity**						
No	29.2	1536.0	REF		REF	
Yes	39.2	676.0	1.56 (1.29–1.89)	0.000	1.34 (1.07–1.68)	0.011
**LGBT School bullying policy**						
No	35.8	522.0	REF		REF	
Yes	24.7	247.0	0.59 (0.42–0.83)	0.002	0.66 (0.45–0.97)	0.036
Don't Know	32.2	1443.0	0.85 (0.69–1.05)	0.135	1.05 (0.82–1.34)	0.714
**Supportive LGBT alliance at educational institution**						
No	36.8	1102.0	REF		REF	
Yes	26.6	331.0	0.62 (0.47–0.82)	0.001	0.66 (0.48–0.91)	0.011
Don't Know	28.3	780.0	0.68 (0.56–0.83)	0.000	0.77 (0.61–0.97)	0.024
**Heard negative remarks regarding sexuality even if was not directed at you**						
Never	27.5	847.0	REF		REF	
Rarely	34.0	632.0	1.36 (1.09–1.70)	0.007	1.33 (1.04–1.70)	0.021
Sometimes	35.2	631.0	1.43 (1.15–1.79)	0.002	1.30 (1.00–1.69)	0.051
Frequently	42.5	106.0	1.94 (1.29–2.94)	0.002	1.54 (0.95–2.50)	0.082

Participants who reported experiencing verbal, physical, or sexual harassment based on their sexuality or gender identity in the past 12 months had increased odds of suicidal ideation (AOR = 1.41, 95 % CI: 1.07–1.86, p = 0.013), as did those who felt deliberately excluded (AOR = 1.57, 95 % CI: 1.05–2.36, p = 0.029). Feeling uncomfortable in an educational setting due to one's sexuality or gender identity was also associated with higher odds of suicidal ideation (AOR = 1.34, 95 % CI: 1.07–1.68, p = 0.011).

Participants who reported hearing negative remarks about sexuality, even if not directed at them, had higher odds of experiencing suicidal ideation. Participants who heard such remarks “rarely” had 34 % higher odds (AOR = 1.33, 95 % CI: 1.04–1.70, p = 0.021), those who heard them “sometimes” had 30 % higher odds (AOR = 1.30, 95 % CI: 1.00–1.69, p = 0.051), although this did not reach significance.

The presence of specific school-based LGBT resources was associated with a reduced likelihood of suicidal ideation among participants. Participants whose schools had an LGBT bullying policy had 34 % lower odds of reporting suicidal ideation (AOR = 0.66, 95 % CI: 0.45–0.97, p = 0.036). Additionally, those with access to a supportive LGBT alliance or club at their educational institution were 34 % lower odds of experiencing suicidal ideation (AOR = 0.66, 95 % CI: 0.48–0.91, p = 0.011).

### 6.2 Suicide attempts in the past 12 months

Table 3 shows the multiple regression analyses for suicide attempt in the past 12 months. Compared to cisgender men, cisgender women had three times higher odds of recent suicide attempt (AOR = 3.48, 95 % CI: 1.71–7.12, p = 0.001). Participants who had experienced verbal, physical, or sexual harassment based on their sexuality or gender identity in the past 12 months had 73 % higher odds of a recent suicide attempt compared to those who did not experience such harassment (AOR = 1.73, 95 % CI: 1.12–2.69, p = 0.014). Similarly, those who reported feeling deliberately left out or excluded due to their sexuality or gender identity in the past 12 months had twice the odds of attempting suicide in the past year (AOR = 2.11, 95 % CI: 1.14–3.90, p = 0.018).

**Table 3:** Regression results for suicide attempt in past 12 months (N = 1997).

	Suicide attempt		Univariable regression		Multivariable regression	
Variable	Number (n)	(%)	OR (95 % CI)	P-value	AOR (95 % CI)	P-value
**Age group (years)**						
15–17	51	15.5	REF		REF	
18–20	94	7.5	0.44 (0.31–0.64)	0.000	0.90 (0.46–1.78)	0.771
21–25	40	7.8	0.46 (0.30–0.72)	0.001	0.88 (0.40–1.95)	0.749
**Sexual orientation**						
Gay	42	6.9	REF		REF	
Lesbian	33	11.1	1.70 (1.05–2.74)	0.030	0.55 (0.23–1.30)	0.172
Bisexual	50	9.8	1.47 (0.96–2.26)	0.077	0.63 (0.29–1.33)	0.223
Pansexual	22	11.5	1.77 (1.03–3.05)	0.040	0.72 (0.30–1.73)	0.465
Heterosexual	7	5.6	0.80 (0.35–1.82)	0.595	0.33 (0.10–1.07)	0.065
Asexual	5	7.4	1.08 (0.41–2.83)	0.877	0.50 (0.16–1.54)	0.226
Unsure	12	9.5	1.43 (0.73–2.80)	0.296	0.73 (0.27–1.94)	0.530
Something else	14	9.1	1.36 (0.72–2.56)	0.341	0.56 (0.22–1.45)	0.230
**Gender identity**						
Cisgender man	51	6.0	REF		REF	
Cisgender woman	134	10.8	1.89 (1.35–2.65)	0.000	3.48 (1.71–7.12)	0.001
**Ethnic background**						
Japanese	170	8.7	REF			
Other/mixed	15	10.7	1.26 (0.72–2.20)	0.417		
**Current educational setting**						
secondary school (high school)	65	16.0	REF		REF	
university	95	7.1	0.41 (0.29–0.57)	0.000	0.66 (0.33–1.34)	0.250
2-year university/vocational school	14	6.1	0.34 (0.19–0.63)	0.000	0.45 (0.19–1.07)	0.069
Other	9	7.2	0.41 (0.20–0.85)	0.016	0.54 (0.21–1.37)	0.194
**Currently engaged in employment**						
No	77	11.2	REF		REF	
yes	107	7.6	0.65 (0.48–0.89)	0.007	0.83 (0.55–1.25)	0.363
**Family or household religious**						
No	150	8.4	REF		REF	
Yes	35	11.3	1.39 (0.94–2.05)	0.098	1.38 (0.90–2.13)	0.141
**Residential location: base Capital city, inner suburban**						
Kanto	72	8.1	REF		REF	
Chubu	23	8.7	1.09 (0.67–1.77)	0.742	0.99 (0.59–1.67)	0.967
Chugoku & Shikoku	11	7.3	0.89 (0.46–1.71)	0.716	0.88 (0.44–1.74)	0.710
Hokkaido	10	14.7	1.97 (0.97–4.02)	0.062	1.36 (0.52–3.55)	0.529
Kansai	34	8.8	1.09 (0.71–1.66)	0.707	1.17 (0.75–1.84)	0.489
Kyushu & Okinawa	15	10.6	1.35 (0.75–2.43)	0.317	1.10 (0.60–2.02)	0.749
Tohoku	11	9.9	1.21 (0.62–2.35)	0.578	1.25 (0.61–2.56)	0.538
**Disclosed sexuality or gender identity to family**						
None	87	8.2	REF		REF	
a few/some	44	10.2	1.27 (0.87–1.87)	0.212	1.09 (0.70–1.69)	0.707
most/all	18	13.1	1.69 (0.98–2.91)	0.057	1.80 (0.94–3.43)	0.075
Not applicable	35	7.5	0.91 (0.61–1.37)	0.652	0.71 (0.39–1.29)	0.261
**Disclosed sexuality or gender identity to friends**						
None	33	8.0	REF		REF	
a few/some	106	8.4	1.06 (0.70–1.59)	0.791	0.97 (0.60–1.58)	0.916
most/all	25	13.0	1.70 (0.98–2.96)	0.058	1.51 (0.75–3.01)	0.247
Not applicable	20	8.6	1.08 (0.60–1.93)	0.793	1.79 (0.78–4.13)	0.172
**Any verbal, physical or sexual harassment in past 12 months based on sexuality or gender identity**						
No	134	7.7	REF		REF	
yes	51	15.0	2.12 (1.50–2.99)	0.000	1.73 (1.12–2.69)	0.014
**Felt deliberately left out or excluded in past 12 months based on sexuality or gender identity**						
No	158	8.0	REF		REF	
yes	25	19.7	2.80 (1.76–4.47)	0.000	2.11 (1.14–3.90)	0.018
**Feel uncomfortable at educational setting due to sexuality or gender identity**						
No	109	7.5	REF		REF	
Yes	74	11.6	1.61 (1.18–2.19)	0.003	1.24 (0.84–1.83)	0.277
**LGBT School bullying policy**						
No	53	10.8	REF		REF	
Yes	14	6.1	0.54 (0.29–1.00)	0.049	0.60 (0.30–1.20)	0.149
Don't Know	114	8.3	0.75 (0.53–1.06)	0.105	1.08 (0.72–1.61)	0.711
**Supportive LGBT alliance at educational institution**						
No	112	10.8	REF		REF	
Yes	21	6.7	0.59 (0.36–0.96)	0.033	0.80 (0.46–1.40)	0.433
Don't Know	50	6.8	0.60 (0.42–0.85)	0.004	0.82 (0.54–1.24)	0.351
**Heard negative remarks regarding sexuality even if was not directed at you**						
Never	49	6.2	REF		REF	
Rarely	54	9.0	1.51 (1.01–2.25)	0.045	1.38 (0.89–2.14)	0.153
Sometimes	63	10.6	1.81 (1.23–2.68)	0.003	1.84 (1.17–2.90)	0.008
Frequently	17	17.0	3.12 (1.72–5.67)	0.000	2.33 (1.05–5.18)	0.039

Negative language surrounding sexuality significantly increased the risk of suicide attempts. Participants who reported sometimes hearing negative remarks about sexuality, even if not directed at them, had 84 % higher odds of a recent suicide attempt compared to those who did not encounter such language (AOR = 1.84, 95 % CI: 1.17–2.90, *p* = 0.008). Those who frequently heard negative language about sexuality faced more than double the odds of a recent suicide attempt (AOR = 2.33, 95 % CI: 1.05–5.18, *p* = 0.039).

## 7 Discussion

The findings of this study offer important insights into the factors associated with suicidal ideation and suicide attempts among LGB+ youth in Japanese educational settings. Consistent with global research, participants reported high levels of suicidal ideation (32.3 %) and suicide attempt (8.8 %) in the past 12 months. These rates are comparable to those observed among LGB+ youth in Australian educational settings, as noted in the foundational study (Hill et al., 2022), and are consistent with the limited data available on LGBT youth in Japan (ReBit, 2022). This study underscores the critical role of both risk factors, such as harassment and exclusion based on sexual orientation or gender identity, and protective factors, including LGBT-supportive bullying policies and LGBT clubs or alliances, in their associations with suicidal ideation and suicide attempts among LGB+ students. These findings highlight the potential of affirming school environments to mitigate suicide risk in this vulnerable population and underscore the urgent need for frameworks and policies that actively reduce harassment and bullying.

LGB+ students who had experienced harassment related to their sexuality or gender identity were considerably more likely to have experienced suicidal ideation and suicide attempts in the past 12 months, aligning with international research linking sexuality and gender identity-based discrimination and victimization with increased suicidality among sexual minority youth (Balakrishnan et al., 2022; Ferlatte et al., 2015; Gorse, 2022; Hatzenbuehler et al., 2013; Hill et al., 2022; Salway et al., 2018). Exclusion based on sexuality or gender identity was also a strong risk factor, with excluded students reporting twice the likelihood of suicide attempts and two-thirds higher odds of suicidal ideation. In Japan, where cultural norms prioritize group conformity, this exclusion may intensify feelings of marginalization and isolation among LGB+ people, further compounding minority stress and vulnerability (Kazama & Kawaguchi, 2010; McLelland, 2000).

The only geographic region that showed a significant association with suicidal ideation was *Chugoku* and *Shikoku*. This finding is consistent with broader demographic and sociological patterns. *Tottori* and *Shimane*, two of Japan's most rural and least populous prefectures, are located in this region. These areas experience high rates of youth outmigration and aging, contributing to reduced access to LGBTQ+ peer networks and affirming services. They were also the prefectures with some of the highest proportion of AIDS to HIV ratios for the majority of the history of Japanese surveillance (National Institute of Infectious Diseases, 2016), indicating the lack of services and knowledge for HIV testing (which is a concentrated epidemic among men who have sex with men (MSM) in Japan) and potential stigma towards sexual and gender minorities. Moreover, compared to urban centres like Tokyo or Osaka, *Chugoku* and *Shikoku* have fewer community resources and healthcare providers with expertise in LGBTQ+ issues. These cultural dynamics may heighten minority stress and reduce opportunities for safe disclosure or peer support. These combined structural, cultural, and policy-related factors may explain the elevated risk observed in this region.

Hearing negative remarks about LGBTQA+ people, even indirectly, was linked to higher risks of suicidal ideation and attempts. Students who encountered such remarks occasionally reported significantly higher suicidal ideation and suicide attempts in the past 12 months, suggesting that exposure to negative language reinforces internalized stigma and increases vulnerability to suicidal thoughts and behaviours (Birkett et al., 2009; Hatzenbuehler, 2011). Likewise, feeling uncomfortable in school due to sexuality or gender identity was strongly associated with suicidal ideation, highlighting the relationship between a non-accepting school climate and mental health (Birkett et al., 2009; Hatzenbuehler, 2011; Kosciw et al., 2009). Together, these findings underscore the necessity of creating affirming educational environments to counteract the damaging effects of societal stigma and negative attitudes associated with increased suicidality among LGB+ youth in educational settings in Japan (Amos et al., 2023; Birkett et al., 2009; Hatzenbuehler, 2011).

Conversely, this study revealed the protective role of school-based support measures. Access to LGBT-inclusive anti-bullying policies and LGBT-supportive clubs was associated with lower odds of suicidal ideation. Specifically, students with access to an LGBT-supportive club had 31 % reduced odds of reporting suicidal ideation, while those in schools with LGBT-inclusive bullying policies had 32 % lower odds of experiencing suicidal thoughts in the past 12 months. These protective factors align with research showing that LGBT-inclusive bullying policies and LGBT alliances foster resilience, peer support, and a sense of belonging, all of which buffer against minority stress and are associated with better mental health (Amos et al., 2023; Gorse, 2022; Hatzenbuehler & Keyes, 2013; Lessard et al., 2020a, 2020b). In Japan, where only 15.0 % of participants reported having a supportive club for LGBT students and 11.1 % reported an LGBT-inclusive anti-bullying policy at their educational institution, there is substantial room to expand such initiatives. Compared to other developed countries with more established school-based LGBT support structures, these figures highlight a critical gap in support for LGB+ youth in Japan.

This study represents a significant step forward in understanding the mental health risks and protective factors associated with suicidality among LGB+ students in Japan. As the first study to explore these associations within the Japanese educational context, it highlights the urgent need for school-based support structures to address the unique mental health challenges faced by LGB+ youth. Findings emphasize that inclusive policies, supportive LGBT alliances, and efforts to reduce sexuality and gender identity-based bullying and harassment are critical to fostering safer and more supportive environments for LGB+ youth in education settings. Given Japan's record high youth suicide rates and the limited visibility of LGB+ issues in educational institutions, these findings support the development of targeted interventions aimed at reducing mental health disparities among LGB+ students.

To address these challenges, policymakers, educators, and mental health professionals in Japan should consider culturally sensitive interventions that reflect its unique sociocultural context. Expanding access to LGBT-inclusive mental health resources, strengthening support for LGBT-friendly clubs and alliances, and implementing anti-bullying policies that explicitly protect sexual minorities are essential steps in mitigating suicidality among LGB+ youth. Notably, while such school-based supports were associated with lower odds of suicidal ideation, none were associated with lower odds of suicide attempt. By contrast, sexuality and gender-identity based harassment and exclusion were associated with higher odds of suicide attempt. As this is a cross-sectional study, these findings reflect associations rather than causal effects, but they suggest that existing school-based supports may be more effective in preventing suicidal ideation than in addressing the acute factors contributing to suicide attempts. This highlights the importance of linking school environments with crisis supports and external mental health services. In Japan, this could include targeted training for school nurses and counsellors, improved referral systems, and the development of culturally competent sexual and gender minority youth-specific suicide prevention resources. Given Japan's high youth suicide rates, fostering educational environments that promote acceptance and belonging, while also addressing acute suicide risk, offers a critical opportunity to improve the well-being of LGB+ youth nationwide.

## 8 Limitations

This study has several limitations that should be considered. First, as data were collected through an online survey, the findings may not fully represent all LGB+ students in Japan. Populations without reliable internet access or those less engaged online may be underrepresented. While young people in Japan are generally highly digitally connected, some may not have exclusive access to internet-enabled devices, and privacy concerns, particularly among those who have not disclosed their sexuality or gender identity, might have prevented some from participating. Nonetheless, as the majority of participants in this study had not disclosed their sexuality or gender identity to their families but still chose to complete the survey, it appears that privacy concerns did not pose a significant barrier for many.

The study employed a convenience sampling method, commonly used in research on hard-to-reach or stigmatized populations such as sexual minorities and substance users (Armstrong et al., 2015; Balakrishnan et al., 2022; Smith et al., 2016; Hill, Amos, et al., 2023). Although this approach limits generalizability, similar high levels of suicidal ideation and suicide attempts have been observed among LGB+ youth in both Japanese and international studies, supporting the relevance of our findings (Hill et al., 2022; ReBit, 2022). Despite these limitations, this study represents the largest survey to date of LGB+ students in Japan, offering important insights into this population's mental health challenges.

Although we refer to *risk* and *protective* factors in this manuscript, these terms are used to describe statistical associations observed in cross-sectional data, and should not be interpreted as evidence of causality or as predictors of future suicidal behaviour.

Finally, as this study relied on self-reported behaviours and attitudes, it is susceptible to social desirability bias, particularly among male participants who may underreport specific behaviours (Kelly et al., 2013). However, the anonymous nature of this online survey likely mitigates such biases compared to other formats, enhancing the reliability of participant responses.

## 9 Conclusion

This study represents an important step in identifying risk and protective factors associated with suicidality among LGB+ students in Japan, underscoring the critical role of LGBTQ-inclusive school bullying policies, support structures and affirming educational environments. In Japan, where group conformity is highly valued, exclusion and harassment are particularly salient challenges for LGB+ youth. By addressing these risk factors and promoting protective measures within educational settings through initiatives like LGBT-supportive clubs and inclusive anti-bullying policies, Japanese policymakers and educators can help reduce mental health disparities among LGB+ youth. Establishing safe and supportive educational environments may help reduce the heightened suicide risk faced by this vulnerable population.

## CRediT authorship contribution statement

**Adam O. Hill:** Writing – review & editing, Validation, Project administration, Investigation, Formal analysis, Conceptualization, Writing – original draft, Software, Methodology, Funding acquisition, Data curation. **Noriyo Kaneko:** Supervision, Methodology, Funding acquisition, Writing – review & editing, Resources, Investigation, Conceptualization. **Natalie Amos:** Writing – review & editing, Investigation, Conceptualization, Writing – original draft, Formal analysis. **Adam Bourne:** Writing – review & editing, Methodology, Funding acquisition, Project administration, Investigation, Conceptualization. **Masachika Yamashiro:** Methodology, Writing – review & editing, Investigation, Conceptualization. **Jami Jones:** Writing – review & editing, Methodology, Conceptualization, Writing – original draft, Funding acquisition. **Stuart Gilmour:** Writing – review & editing, Supervision, Methodology, Funding acquisition, Writing – original draft, Project administration, Investigation, Conceptualization.

## Ethical statement

Ethical approval for the *Writing Themselves in Japan* (WTIJ) study was obtained from the Human Research Ethics Committee of St Luke's International University. All participants provided informed consent prior to participation. For participants under the age of 18, parental consent was waived by the ethics committee due to the minimal risk nature of the research and the importance of ensuring participant safety and confidentiality. All procedures were carried out in accordance with the ethical standards of the institutional and national research committees, and with the 1964 Helsinki Declaration and its later amendments or comparable ethical standards.

## Funding

This research was supported by funding from the Japan Society for the Promotion of Science (JSPS) Fellowship and a KAKEN grant provided by the Japan Society for the Promotion of Science (Grant Number: 22KF0339.) It was further supported by Grant-in-Aid for Scientific Research (KAKENHI) (B) (Grant Number: 23H03233). The authors are grateful for this support, which facilitated the design, implementation, and analysis of the Writing Themselves in Japan (WTIJ) survey. The funders had no role in the study design, data collection, analysis, interpretation of results, or the writing of this manuscript.

## Acknowledgements

This research was made possible through the input and support of community and youth advisory board members, as well as individuals in Australia and Japan with lived experience who generously shared their expertise. We are especially grateful to the young LGBTQA+ participants who gave their time to complete the survey. We also thank the many young people with lived experience who provided feedback and participated in survey testing. We are grateful to the peer reviewers and to Ms Yoshikawa for their valuable input. Finally, this paper is dedicated to Ivy McGowan, whose life and legacy inspired this research and continue to be remembered with the deepest appreciation and affection.

## Declaration of interest statement

The authors declare no conflicts of interest.

## Data Availability

The data that has been used is confidential.
